# Rare Coexistence of Ménétrier’s Disease and Gallbladder Duplication in a Young Male Adult: A Case Report

**DOI:** 10.3390/diagnostics16172867

**Published:** 2026-09-07

**Authors:** Irina Ciortescu, Roxana Nemteanu, Otilia Nedelciuc, Mihaela Dranga, Radu Sebastian Gavril, Andrei Olteanu, Andreea Clim, Elena-Lavinia Mujdei, Alexandru Ionut Coseru, Alina Plesa

**Affiliations:** 1 Faculty of Medicine, “Grigore T. Popa” University of Medicine and Pharmacy, 700115 Iasi, Romania; irinaciortescu@yahoo.com (I.C.); otilianedelciuc@yahoo.com (O.N.); mihaela_dra@yahoo.com (M.D.); rgavril87@yahoo.com (R.S.G.); olteanuandrei@yahoo.com (A.O.); andreea.clim13@yahoo.com (A.C.); ionut_ionut_barlad@yahoo.com (A.I.C.); alinaplesaro@yahoo.com (A.P.); 2 Institute of Gastroenterology and Hepatology, “Saint Spiridon” Hospital, 700115 Iasi, Romania; lavinia.juncanariu@gmail.com; 3 Department of Internal Medicine, “Grigore T. Popa” University of Medicine and Pharmacy, 700115 Iasi, Romania

**Keywords:** Ménétrier’s disease, gallbladder duplication, *Helicobacter pylori*

## Abstract

**Background and Objectives:** Ménétrier’s disease (MD) is an exceptionally rare hypertrophic gastropathy characterized by foveolar hyperplasia, gastric acid suppression, and protein-losing enteropathy. Congenital gallbladder duplication is a rare biliary anomaly associated with independent pathological risks and surgical complications. **Case Presentation:** We report the case of a 34-year-old male presenting with chronic epigastric and right hypochondriac pain, alongside persistent, uninvestigated polycythemia. Upper endoscopy and histopathology revealed diffuse foveolar hyperplasia with cystic oxyntic gland dilatation and active *Helicobacter pylori* infection, confirming MD. Magnetic resonance cholangiopancreatography demonstrated a double gallbladder with independent cystic ducts. Successful *H. pylori* eradication was achieved, and hematological workup ruled out primary myeloproliferative neoplasm. **Conclusions:** To our knowledge, this is the first reported case of concurrent MD, and double gallbladder. This report underscores the necessity of a systematic diagnostic approach combining advanced imaging and histopathology to manage complex, overlapping abdominal pathologies.

## 1. Introduction

Ménétrier’s disease (MD) is an enigmatic, rare gastropathy defined by remarkable gastric hypertrophy with an undefined global prevalence, primarily due to the paucity of large-scale epidemiological studies [1]. It is characterized by the diffuse proliferation of foveolar epithelial cells. This foveolar overgrowth is accompanied by glandular atrophy, decreased gastric acid secretion (hypo- or achlorhydria), and massive loss of serum proteins across the damaged gastric barrier (protein-losing enteropathy), which clinically manifests as hypoalbuminemia and peripheral edema [2]. The condition predominantly manifests in adults, typically during the fourth to sixth decades of life, with a notable male preponderance. The more recent scoping reviews indicate an increasing recognition of the disease as endoscopic imaging improves [1,3].

Although more than a century has passed since its initial description by Pierre Ménétrier in 1888 [4], the precise etiology of MD remains incompletely understood. In the adult population, MD is characterized by an insidious and progressive course, and it is now strictly categorized as a premalignant condition [5]. Long-term follow-up is recommended, as an increased risk of gastric adenocarcinoma has been documented in adult patients with MD, necessitating rigorous endoscopic surveillance [6]. The exact pathogenesis of MD remains elusive, though it is widely considered a multi-factorial process. It has been etiologically linked to infectious triggers, most notably *Helicobacter pylori* (*H. pylori*) and Cytomegalovirus (CMV), yet some cases have no connection to any known infectious causes [7,8].

Congenital anomalies of the biliary system, though slightly more common, still pose major clinical and radiological challenges [9]. Gallbladder duplication (double gallbladder) is a rare anatomical variant resulting from an abnormal division of the gallbladder primordium during embryogenesis. Its reported prevalence is approximately 1 in 4000 individuals (0.015% to 0.03%). It is classified based on the relationship of the gallbladder bodies and their cystic duct drainages [10,11].

To our knowledge, the simultaneous occurrence of MD and a congenital double gallbladder has not been previously reported in the literature. In this study, we present the clinical management, histopathological features, imaging markers, and multi-specialty diagnostic strategy for a 34-year-old male presenting with overlapping upper abdominal pain due to these coexisting rare entities.

## 2. Case Presentation

A 34-year-old male IT specialist presented with a multi-week history of worsening, recurrent upper abdominal pain localized to the epigastrium and right hypochondrium. The patient had made multiple recent emergency department (ED) visits for acute pain control. His past medical history was notable for persistent, uninvestigated polycythemia with documented hemoglobin levels consistently exceeding 18 g/dL over several years. To systematically evaluate potential secondary causes of absolute erythrocytosis, a detailed clinical history was taken: the patient was a non-smoker, had no history of chronic obstructive pulmonary disease or sleep apnea, maintained normal resting oxygen saturation on room air >98%, and reported no exposure to erythropoiesis-stimulating agents, exogenous testosterone, or anabolic steroids. He reported occasional alcohol consumption, and had no family history of gastrointestinal malignancies or systemic diseases. On physical examination, the patient appeared moderately distressed due to persistent abdominal discomfort. He had a normal body mass index, non-icteric skin and mucus membranes, and no peripheral edema. Cardiovascular, respiratory, and genitourinary examinations were unremarkable. Abdominal examination revealed a soft, non-distended abdomen with severe tenderness on deep palpation in the epigastrium and right hypochondrium, without peritoneal signs.

Initial laboratory workup showed increased hemoglobin levels of 18.4 g/dL and a hematocrit of 50.8%. Interestingly, serum biochemistry highlighted low total proteins (4.84 g/dL) and borderline-low albumin (3.05 g/dL). Tumor markers (CEA, CA 19-9, AFP) and viral hepatitis markers were negative. The main laboratory parameters are summarized in Table 1.

**Figure 1:**
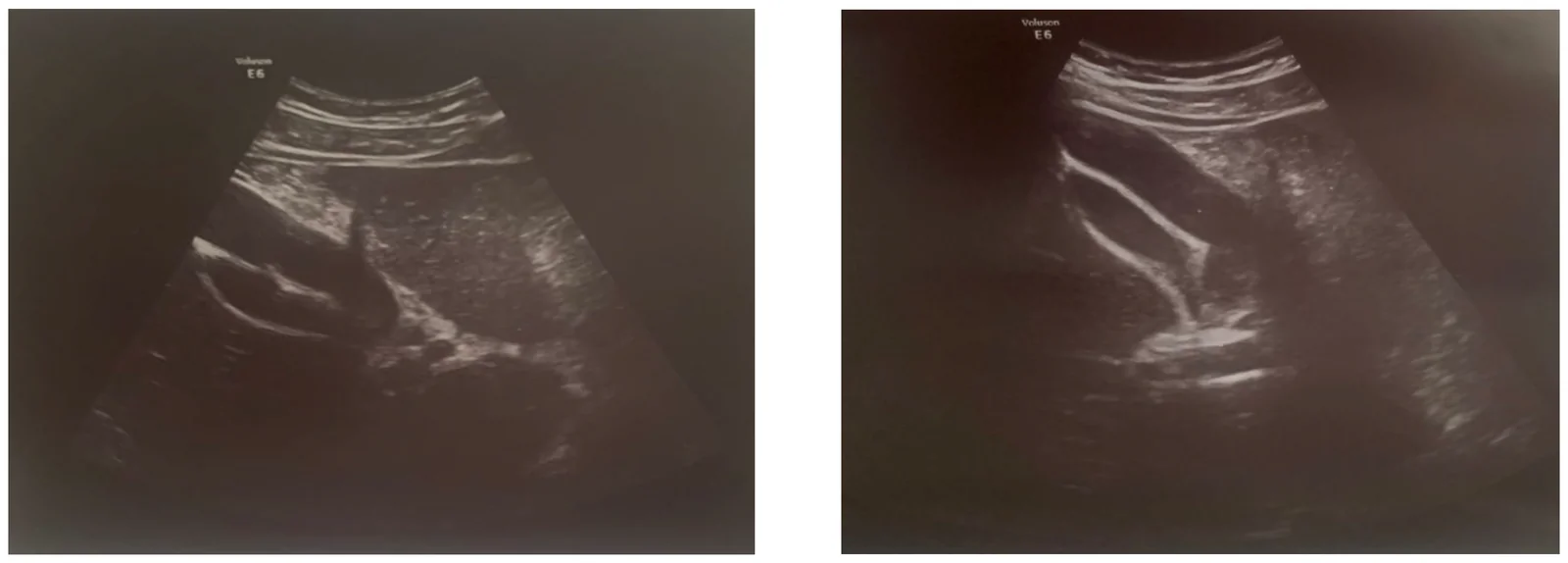
Transabdominal ultrasound demonstrating a duplicated, dual-chambered gallbladder without wall thickening or intraluminal cholelithiasis.

Abdominal ultrasonography demonstrated a normal liver parenchyma without focal lesions and normal portal vein caliber. Biliary evaluation revealed a dual-chambered double gallbladder without intraluminal lithiasis or wall thickening (Figure 1). The pancreas and kidneys were unremarkable. The spleen was moderately enlarged, measuring 150 mm in its longest longitudinal axis.

**Figure 2:**
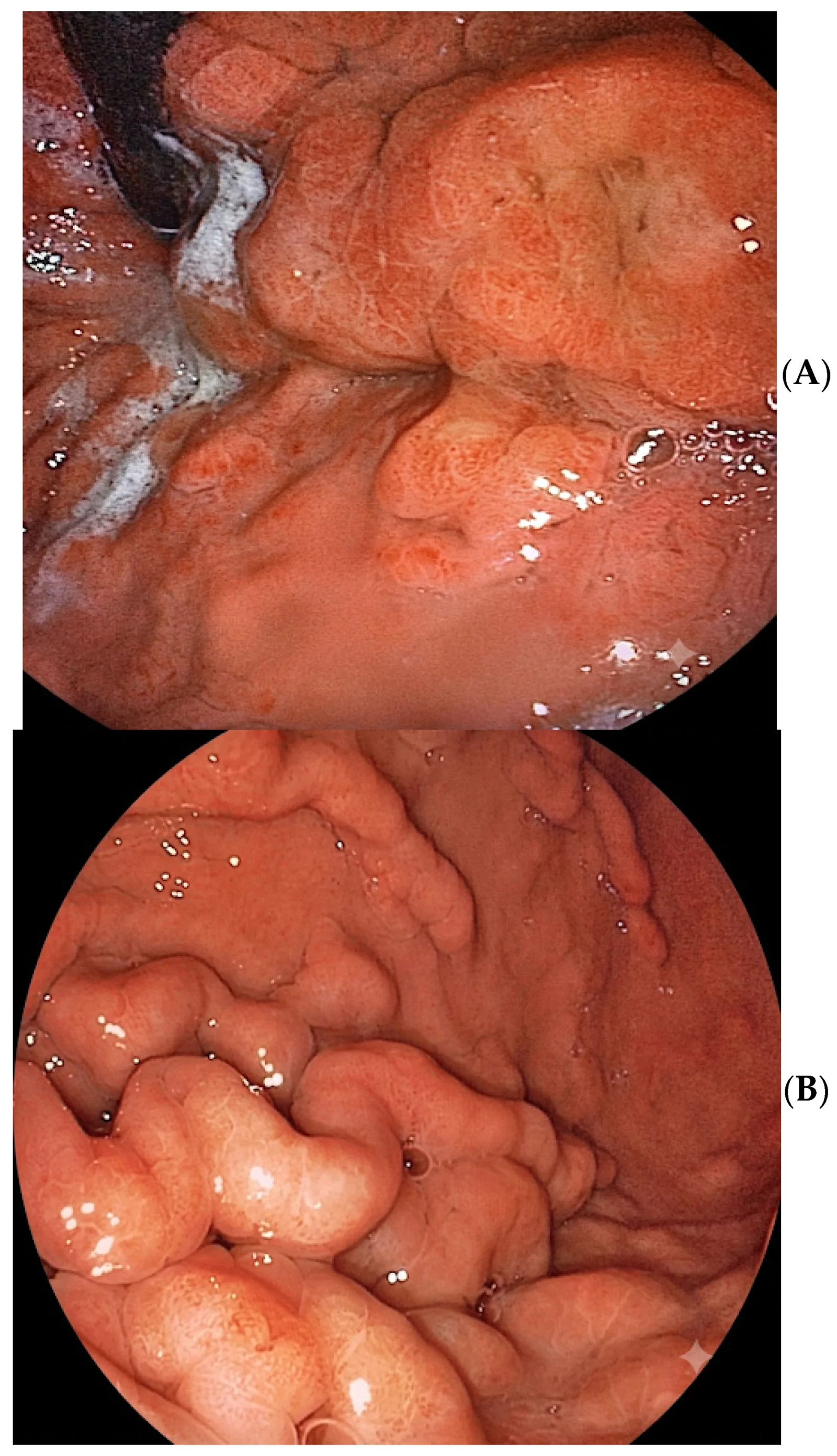
EGD showing prominent, giant pseudotumoral mucosal folds in the fundus (**A**) and gastric body (**B**) covered with thick mucus.

**Figure 3:**
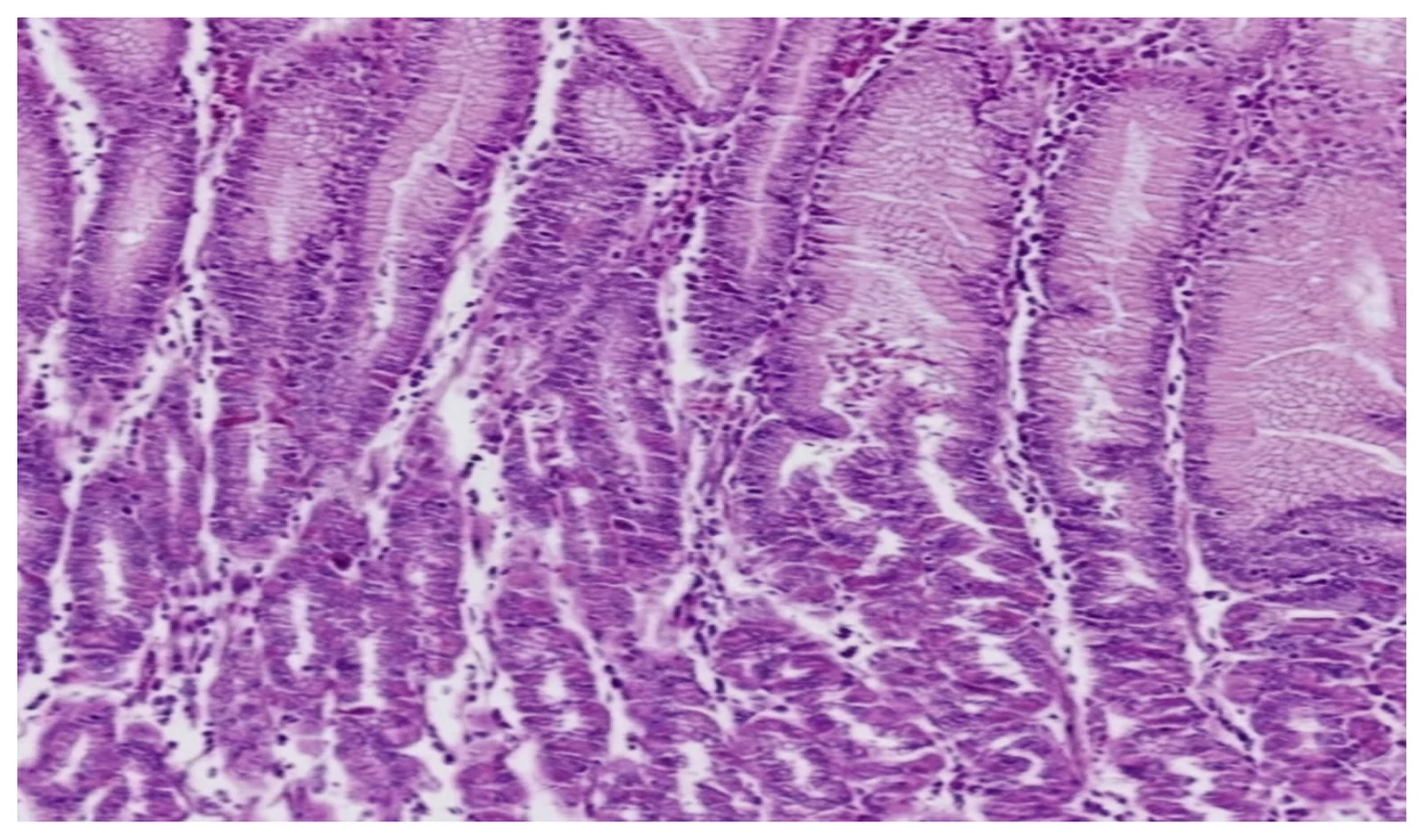
Marked elongation and tortuosity of foveolar epithelium, chronic inflammatory cell infiltrate in the lamina propria.

Upper gastrointestinal endoscopy (EGD) demonstrated diffuse, giant pseudotumoral mucosal folds in the gastric body and fundus covered with abundant, thick mucus (Figure 2). To ensure adequate architectural evaluation beyond superficial epithelium, multiple deep mucosal biopsies were obtained from the hyperplastic folds from the fundus and gastric body using jumbo biopsy forceps via a bite-on-bite technique. Histopathological examination of the full-thickness mucosal samples confirmed marked foveolar hyperplasia with elongated, tortuous foveolar crypts and cystic glandular dilatation, accompanied by prominent parietal cell and oxyntic glandular atrophy (Figure 3). Giemsa, Masson’s trichrome and Alcian Blue (pH 2.5)/PAS staining were employed and identified abundant *H. pylori* organisms. Ki-67 proliferation index was <5% within the stroma and restricted strictly to the foveolar stem cell niche. Fasting serum gastrin levels and gastric pH monitoring were not obtained prior to the initiation of empirical proton pump inhibitor (PPI) therapy in the emergency setting, representing a clinical limitation.

There was no evidence of intestinal metaplasia or high-grade dysplasia. The histopathological features were fully consistent with chronic foveolar hypertrophic gastropathy–MD associated with active *H. pylori* infection.

The definitive diagnosis of MD in this patient was established by integrating key clinical, biochemical, endoscopic, and histopathological criteria:

- **Clinical:** Severe, recurrent epigastric discomfort leading to repeated emergency evaluations.
- **Biochemical:** Significant hypoproteinemia (total protein: 4.84 g/dL) with borderline-low serum albumin (3.05 g/dL), reflecting subclinical protein-losing enteropathy without peripheral edema.
- **Endoscopic:** Prominent pseudotumoral giant mucosal folds confined to the gastric body and fundus, sparing the antrum, accompanied by hypersecretion of thick, viscous mucus.
- **Histopathological:** Bite-on-bite deep mucosal biopsies demonstrating classic architectural remodeling characterized by massive foveolar epithelial hyperplasia with elongated, tortuous crypts, cystic glandular dilatation, and associated parietal cell/oxyntic glandular atrophy.

The combination of these characteristic histopathological features with classic endoscopic rugal hypertrophy and biochemical protein depletion effectively ruled out morphologic mimickers, confirming MD.

Since *H. pylori* is strongly linked to the pathogenesis and exacerbation of MD in adults, and successful eradication has been shown to induce complete regression of the gastropathy in selected cases [12], an empiric 14-day first-line eradication regimen was immediately prescribed. The patient was initiated on an *H. pylori* eradication protocol consisting of Levofloxacin 500 mg (1/day), Amoxicillin 2 × 500 mg twice daily, and Esomeprazole 40 mg (1 tab × 2/day) for 14 days, along with albumin infusion and volume replacement therapy with continuation of PPI use up to 8 weeks.

**Figure 4:**
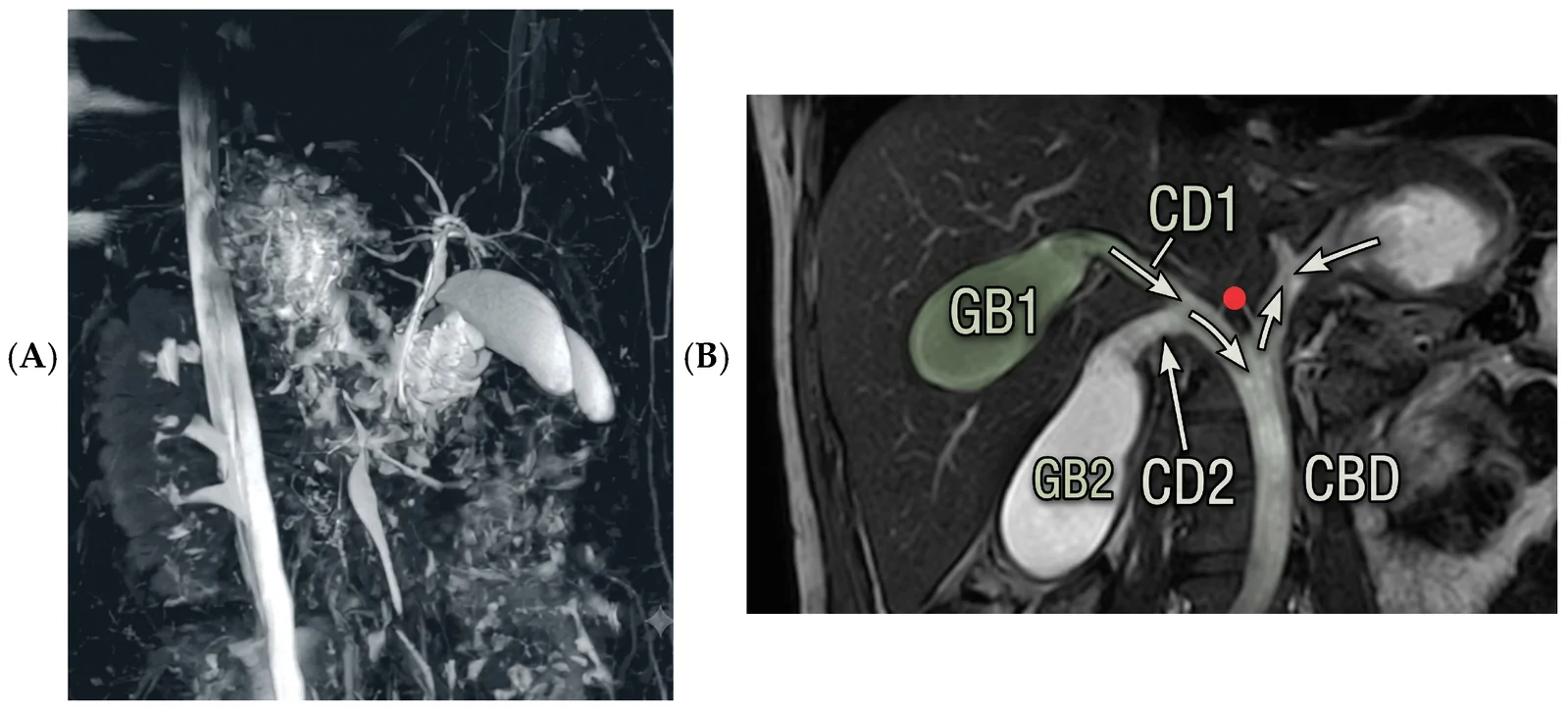
(**A**) MRCP 3D reconstruction confirming gallbladder duplication with distinct cystic ducts; (**B**) two distinct gallbladder lumens: GB1 and GB2; each gallbladder gives rise to its own dedicated drainage canal: cystic duct 1 (CD1) from GB1 and cystic duct 2 (CD2) from GB2; CD1 and CD2 converge medially at a single junction (marked by the red dot) to form a common cystic duct before draining directly into the main CBD.

To map the suspected double gallbladder, magnetic resonance cholangiopancreatography (MRCP) was performed. MRCP demonstrated two well-formed, independent gallbladder bodies residing within the gallbladder fossa. Each gallbladder gave rise to an independent cystic duct. The two cystic ducts converged to form a short common channel prior to inserting into the supraduodenal common bile duct (CBD). This anatomy corresponds to a true gallbladder duplication of the Y-shaped type according to Boyden’s classification (or Harlaftis Type 1, Y-shaped variant) (Figure 4). Neither gallbladder demonstrated gallstones, sludge, or acute inflammatory changes, and the extrahepatic biliary tree showed normal caliber.

A structured, multidisciplinary management plan was established:

1. **Hematological Workup:** Formal hematology consultation.
2. **Eradication Verification:** Urea breath testing, stool antigen or biopsy sample at 6–8 weeks post treatment.
3. **Biochemical Monitoring:** Re-evaluation of total serum proteins and albumin at 4–6 weeks to assess resolution of protein loss.
4. **Endoscopic Surveillance:** Repeat EGD with mucosal biopsies at 6–12 months to assess regression of rugal hypertrophy, and long-term endoscopic surveillance.
5. **Biliary Surveillance:** Annual ultrasound monitoring. As both gallbladders were functional and alithiasic, prophylactic cholecystectomy was not indicated, though surgical consultation was advised should biliary symptoms arise.

**Figure 5:**
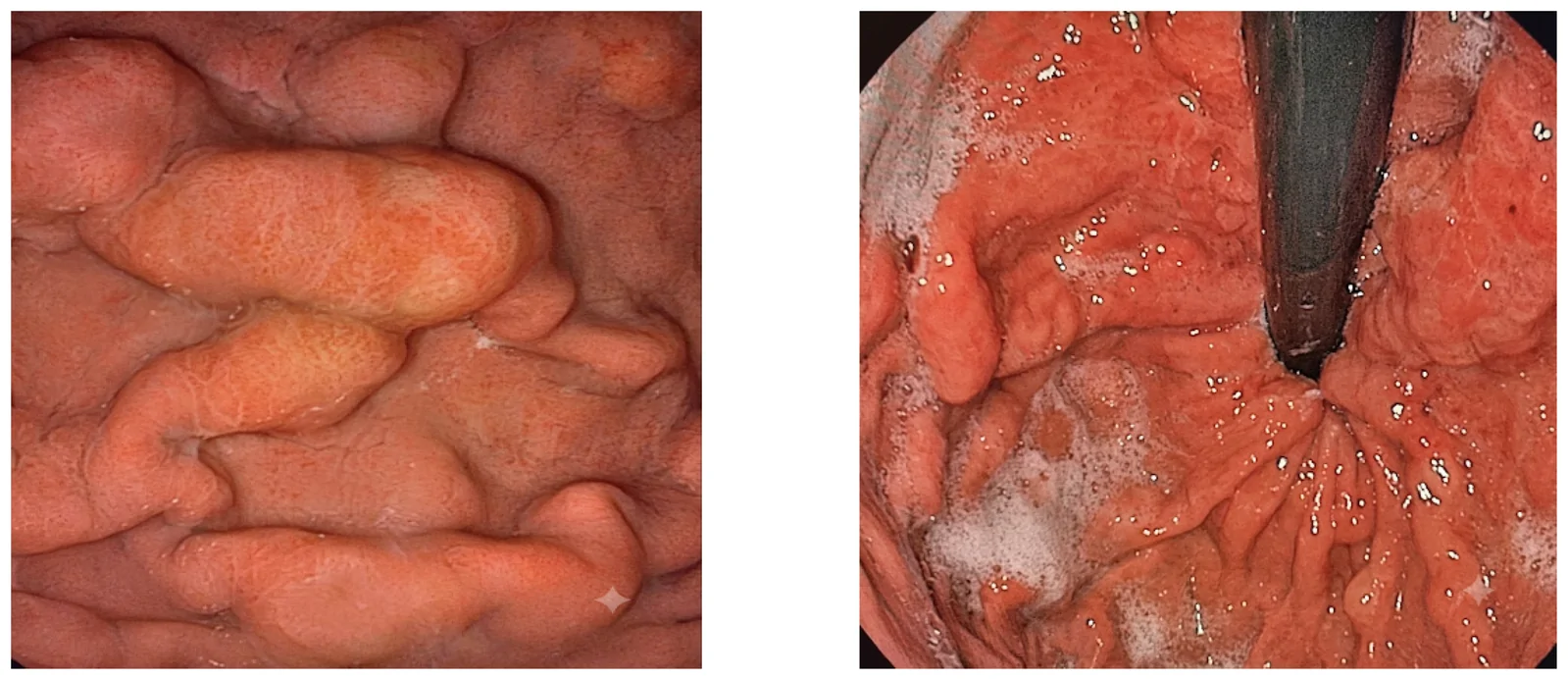
Follow-up EGD at 3 months post *H. pylori* eradication showing persistent enlarged gastric folds despite complete mucosal infection clearance.

At the 3-month follow-up evaluation, a comprehensive multi-modal assessment demonstrated significant clinical and biological response despite persistent mucosal fold enlargement. HEGD demonstrated persistent enlarged, pseudotumoral mucosal folds in the fundus and corpus (Figure 5), though mucosal hyperemic congestion and thick mucus hypersecretion were noticeably diminished. Follow-up deep mucosal biopsies were obtained using jumbo forceps via a bite-on-bite technique from the identical anatomical regions sampled at baseline (specifically the hyperplastic giant folds of the gastric fundus and body). Histopathological examination confirmed complete clearance of *H. pylori* organisms (confirmed by negative Giemsa staining) and a significant reduction in lamina propria chronic inflammatory infiltrate. Foveolar hyperplasia remained present but showed early attenuation of epithelial height. The patient reported complete resolution of epigastric and right hypochondriac pain, with no recurrence of acute emergency visits. Hematological investigation revealed a normal serum erythropoietin level (8.2 mIU/mL; reference range: 4.3–29.0 mIU/mL) and negative test results for the *JAK2* V617F mutation, effectively excluding primary polycythemia vera and autonomous erythropoietin production. Given the complete, sustained normalization of red cell indices (Hb 14.8 g/dL, Ht 43.2%), following intravenous fluid resuscitation, albumin repletion, and mucosal stabilization, the presentation was diagnosed as relative erythrocytosis driven by transient hemoconcentration and plasma volume contraction.

## 3. Discussions

MD is an extremely rare clinical entity in adult gastroenterology, representing a form of hypertrophic gastropathy [13]. Its pathogenesis is characterized by massive foveolar hyperplasia, causing a protein-losing enteropathy and hypoalbuminemia, which classically manifests as anasarca or peripheral edema. However, the clinical spectrum of MD can be variable [14]. In some patients, as observed in our case, protein loss may remain subclinical resulting in borderline-low serum albumin concentrations without peripheral edema and the mucosal enlargement may present as localized, pseudotumoral nodular folds rather than classic diffuse giant rugal hypertrophy [1].

The parallel clinical findings involving *H. pylori* warrant consideration, as the bacterial infection is thought to upregulate TGF-α and epidermal growth factor receptor (EGFR) signaling, driving foveolar hyperplasia. When *H. pylori* infection is identified in patients with MD, eradication therapy is strongly recommended; while complete clinical, endoscopic, or histological remission has been reported in some published cases following successful clearance, this response is variable and complete remission does not occur consistently across all patients [15]. Similarly, CMV infection induces acute injury to the gastric mucosa, resulting in an abnormal local accumulation of TGF-α. Specifically, viral replication within the gastric epithelium has been shown to upregulate intracellular signaling cascades and activate specific proto-oncogenes [8,16]. This molecular environment promotes the sustained expression of TGF-α in gastric mucosal cells, which in turn drives the characteristic expansion of the foveolar compartment while inhibiting the maturation of acid-secreting parietal cells. Other associations cited in the literature include herpes simplex and *Giardia lamblia* infections, rare familial occurrences, autoimmune comorbidities, inflammatory bowel disease, rheumatological and hepatobiliary diseases [17,18].

Although generalized rugal hypertrophy is a shared endoscopic hallmark of hypertrophic gastropathies, MD must be rigorously differentiated from other conditions that mimic giant or pseudotumoral gastric folds [1]. These include Zollinger–Ellison syndrome (ZES), lymphocytic gastritis, primary gastric lymphoma, and gastric involvement in juvenile polyposis syndrome [19]. Pathologically, ZES is characterized by hypergastrinemia-driven expansion of the deeper oxyntic glandular compartment, resulting in an increased parietal cell mass. This contrasts sharply with the superficial foveolar hyperplasia and parietal cell atrophy characteristic of MD. Furthermore, ZES clinically presents with severe gastric hyperacidity and refractory peptic ulceration, whereas MD is defined by hypochlorhydria and protein-losing enteropathy [20]. A recognized limitation of our workup includes the lack of pre-treatment fasting serum gastrin concentrations and formal gastric acid output analysis prior to the administration of high-dose PPIs. Although serum gastrin measured during PPI therapy carries limited diagnostic specificity due to drug-induced hypergastrinemia, the definitive histopathological architecture showing foveolar proliferation alongside parietal cell loss reliably differentiates MD from gastrinoma-driven oxyntic hyperproliferation.

Lymphocytic gastritis is distinguished histologically by a dense accumulation of intraepithelial lymphocytes without the profound foveolar architectural remodeling or protein depletion seen in MD [21]. Primary gastric lymphoma can extensively infiltrate the submucosa and lamina propria, creating massive, rigid rugal folds that closely mimic hypertrophic gastropathy endoscopically; however, it is differentiated by a monomorphic infiltrate of atypical lymphocytes and characteristic lymphoepithelial lesions [22]. Gastric juvenile polyposis syndrome can generate diffuse, confluent hamartomatous polyps that resemble giant folds [23]. Histologically, these are distinguished by an abundance of edematous stroma rather than true foveolar epithelial dominance, and the condition is frequently delineated by a distinct genetic profile or concomitant lower gastrointestinal polyps [24].

In the context of our patient, distinguishing among these entities was crucial given the atypical presentation of the disease. The presence of pseudotumoral rather than classic giant gastric folds necessitated a rigorous differentiation from primary gastric lymphoma and juvenile polyposis, both of which can closely mimic this nodular, mass-like endoscopic appearance. Immunohistochemical profiling, and specific stromal staining parameters were employed to differentiate MD from morphologic mimickers. Furthermore, because the patient’s protein loss was subclinical resulting in borderline-low serum albumin levels without overt peripheral edema, MD could have easily been misdiagnosed as lymphocytic gastritis [1,2]. Ultimately, histopathological confirmation of true superficial foveolar hyperplasia accompanied by glandular atrophy, rather than deep oxyntic expansion, atypical lymphocytic infiltration, or hamartomatous stromal edema, firmly established the diagnosis of MD [25].

Management of MD is tailored to symptom severity and the underlying pathophysiology, focusing primarily on supportive care, targeting infection, and suppressing overactive signaling pathways [1]. Supportive measures center on mitigating protein loss through a high-protein diet, micronutrient replacement, and intravenous albumin infusions for severe hypoalbuminemia [26]. Initial medical management includes eradicating *H. pylori* or treating CMV with ganciclovir when positive, as clearing these infections can significantly improve disease status, alongside PPIs to decrease gastric acid output [27].

Referring to the case presented, because antimicrobial susceptibility testing (culture or molecular PCR) is not routinely available in local practice, an empirical eradication protocol was selection-guided by regional antibiotic resistance patterns. In Eastern Europe and Romania, reported primary clarithromycin resistance consistently exceeds 15–20% [28], making standard clarithromycin-based triple therapy unsuitable for empirical first-line treatment. Due to the temporary hospital unavailability of oral bismuth salts required for classic bismuth quadruple therapy, an empirical 14-day levofloxacin-based triple therapy was selected [29]. In our patient, a 14-day extended regimen successfully achieved *H. pylori* clearance.

The relationship between *H. pylori* and MD remains controversial. Although successful eradication has been followed by symptomatic and endoscopic improvement in published case reports, this observation is not universal. Rigorous case–control data demonstrate no increased prevalence of *H. pylori* in MD patients compared to controls [30]. Therefore, eradication therapy should be viewed as a targeted anti-infective measure rather than a guaranteed disease-modifying cure for MD. As established in long-term observational series of adult-onset MD, cellular turnover and macro-architectural regression of massive foveolar hyperplasia lag behind microbiological cure. Complete architectural flattening of giant rugal folds frequently requires 6 to 18 months following infection clearance. This temporal mismatch highlights why objective biochemical markers and repeat targeted histopathology rather than endoscopic appearance alone must guide short-term treatment response evaluation [1,12].

For targeted disease modification, therapies aimed at the dysregulated TGF-α–EGFR pathway have shown significant promise. The somatostatin analog octreotide modulates the dysregulated TGF-α–EGFR pathway—typically daily or monthly as a long-acting depot—yielding clinical and histological recovery in reported cases [31]. Additionally, cetuximab, a monoclonal EGFR-inhibiting antibody, directly blocks ligand binding to reduce mucosal hypertrophy and protein loss, representing the subject of the sole Phase I clinical trial evaluated for MD to date [32].

Simultaneously, the patient presented with a double gallbladder, a highly unusual embryological developmental anomaly with an incidence of 0.015% to 0.03% [11]. Accurate anatomical categorization relies on recognized surgical and embryological classification systems. In Boyden’s classification, duplicated gallbladders are divided into *vesica fellea divisa* (bilobed/septate) and *vesica fellea duplex* (true duplication with two cystic ducts). True duplication is further subclassified into the Y-shaped type (where two cystic ducts unite into a shared common trunk before entering the CBD) and the H-shaped or ductular type (where two cystic ducts enter the CBD separately at distinct sites) [10]. Our patient presented with a classic Y-shaped duplication. The primary clinical significance of this anomaly lies in the fact that each gallbladder functions independently. Therefore, pathologic processes (such as cholelithiasis, cholecystitis, or neoplasia) can affect one or both gallbladders. Preoperative recognition of this anomaly is vital; if unrecognized, surgeons may perform a cholecystectomy on only one gallbladder, leaving the diseased duplicate behind, or cause serious accidental common bile duct transections during surgery [33]. While ultrasound can easily miss a duplicate or mistake it for a gallbladder diverticulum or folded wall, MRCP represents the non-invasive gold standard diagnostic modality to clearly map the biliary anatomy [34].

The coexistence of MD and congenital double gallbladder is highly unusual. There is no evidence in the medical literature indicating a shared pathogenetical pathway between these two conditions. However, their coexistence creates major diagnostic challenges. The patient’s clinical presentation consisting of both epigastric pain (typical of MD) and right hypochondrium pain (typical of gallbladder disease) created a mixed and confusing abdominal symptom complex. Without careful, stepwise endoscopic and high-resolution radiological examinations (MRCP), either of these rare diagnoses could have been easily missed or misdiagnosed as standard peptic ulcer disease or biliary dyskinesia [35].

An additional notable feature of this case was persistent erythrocytosis accompanied by moderate splenomegaly (150 mm). When evaluating coexisting erythrocytosis in the context of hypertrophic gastropathy, it is crucial to carefully distinguish between temporal association, biological plausibility, and demonstrated causality. The patient’s initial presentation with hemoglobin >18 g/dL raised suspicion for polycythemia vera versus secondary or relative erythrocytosis. Normal serum erythropoietin levels and the absence of a *JAK2* V617F mutation significantly reduced the probability of an underlying myeloproliferative neoplasm (MPN), though complete exclusion of rare MPN variants ideally requires bone marrow histopathology. Because this represents a single clinical observation and the patient reported elevated hemoglobin values predating his acute presentation by several years, a direct causative or molecular linkage between MD and erythrocytosis cannot be established. Intermittent dehydration, lifestyle factors, or baseline physiological variation may have acted as major confounders. The temporal alignment between the resolution of GI symptoms, protein depletion and the normalization of red blood cell counts suggests that mucosal injury, protein-losing enteropathy, and reduced fluid intake induced a state of subclinical plasma volume depletion. The biological plausibility of relative erythrocytosis in MD stems from hemoconcentration caused by severe gastropathy-associated intravascular volume shifts. Therefore, rather than implying a unified pathophysiological driver, the co-occurrence reported here represents a temporal association where gastrointestinal fluid loss and protein enteropathy acute-on-chronically unmasked relative erythrocytosis [7].

Furthermore, regarding the persistence of enlarged mucosal folds at the 3-month follow-up EGD (Figure 5), it is well established that structural mucosal remodeling and architectural regression of giant or pseudotumoral folds following successful *H. pylori* eradication typically require an extended timeline ranging from 6 months to over a year [1]. Long-term follow-up is recommended, as an increased risk of gastric adenocarcinoma has been reported in patients with MD, with one retrospective study estimating a 10-year incidence of 8.9% [30], necessitating rigorous endoscopic surveillance [32].

## 4. Conclusions

This case report documents a unique clinical presentation involving the unprecedented coexistence of MD, congenital double gallbladder and relative erythrocytosis. While a single case cannot establish a definitive mechanistic link between these entities, this presentation underscores the diagnostic complexity and clinical challenges posed by their concurrent occurrence. Rather than indicating a shared pathophysiological driver, this unusual coexistence highlights the paramount importance of a systematic differential diagnosis when evaluating atypical presentations.

## Figures and Tables

**Table 1:** Laboratory parameters at baseline presentation and during follow-up.

Parameter	Baseline Presentation	3-Month Follow-Up	Reference Range
White Blood Cells (GA)	6830/µL	7130/µL	4000–10,000/µL
Hemoglobin (Hb)	18.4 g/dL	14.8 g/dL	13.5–17.5 g/dL (Male)
Hematocrit (Ht)	50.8%	43.2%	40.0–50.0% (Male)
Mean Corpuscular Volume (MCV)	87.0 fL	91 fL	80–100 fL
Mean Corpuscular Hemoglobin (MCH)	32.0 pg	28 pg	27–32 pg
Total Proteins	4.84 g/dL	6.90 g/dL	6.6–8.7 g/dL
Albumin	3.05 g/dL	4.20 g/dL	3.5–5.2 g/dL
C-Reactive Protein (CRP)	0.49 mg/dL	0.35 mg/dL	<0.50 mg/dL
Alanine Aminotransferase (TGP)	37 U/L	33 U/L	<50 U/L
Aspartate Aminotransferase (TGO)	42 U/L	40 U/L	<50 U/L
Gamma-Glutamyl Transferase (GGT)	27 U/L	21 U/L	<60 U/L
Alkaline Phosphatase (FAL)	40 U/L	72 U/L	40–130 U/L
Total Bilirubin	0.94 mg/dL	0.87 mg/dL	<1.2 mg/dL
Direct Bilirubin	0.38 mg/dL	0.21 mg/dL	<0.3 mg/dL
Serum Iron	130 µg/dL	99 µg/dL	59–158 µg/dL
Ferritin	188 µg/dL	201 µg/dL	30–400 µg/dL
Total Cholesterol	131 mg/dL	191 mg/dL	<200 mg/dL
Lipase	51 U/L	19 U/L	10–140 U/L
Clinical Symptoms	Severe, recurrent epigastric and right hypochondriac pain	Complete symptomatic resolution	Asymptomatic
Endoscopic Findings	Diffuse, giant pseudotumoral folds in fundus/body with thick mucus	Persistent enlarged mucosal folds; reduced hyperemic congestion and mucus	Normal mucosal fold architecture
Histopathological Features	Marked foveolar hyperplasia, tortuous crypts, parietal cell atrophy	Persistent foveolar hyperplasia with early epithelial attenuation	Normal gastric architecture
*H. pylori* Status (Giemsa)	Positive (active infection)	Negative (eradicated)	Negative

## Data Availability

The data presented in this study are available on request from the corresponding author.

## References

[1] Barros A.R., Monteiro S., Silva J. (2025). Ménétrier disease: A clinical review. World J. Gastroenterol..

[2] Wang X., Liu H. (2025). Ménétrier’s Disease. N. Engl. J. Med..

[3] Lin W.X., Yang K.Y., Xie W. (2026). Ménétrier Disease. Mayo Clin. Proc..

[4] Ménétrier P. (1888). Des polyadénomes gastriques et de leurs rapports avec le cancer de l’estomac. Arch. Physiol. Norm. Pathol..

[5] Hirsch B.S., Cardoso S.R., Baba E.R., de Moura D.T.H., Gonçalves M.E.P., Rocha R.S.P., de Moura E.G.H. (2023). Chronic Ménétrier disease leading to gastric cancer in youth. Clin. Endosc..

[6] Thammineedi S.R., Rao V.B., Pasam M.K., Saksena A.R., Nusrath S. (2021). Menetrier’s Disease Masquerading as Adenocarcinoma of Stomach. Indian J. Surg. Oncol..

[7] Gore M., Bansal K., Goosenberg E. (2026). Menetrier Disease. StatPearls.

[8] Barbati F., Marrani E., Indolfi G., Lionetti P., Trapani S. (2021). Menetrier disease and Cytomegalovirus infection in paediatric age: Report of three cases and a review of the literature. Eur. J. Pediatr..

[9] Takahashi H., Raman R. (2025). A systematic review of gallbladder anomalies. J. Soc. Laparosc. Robot. Surg..

[10] Boyden E.A. (1926). The accessory gall-bladder—An embryological and comparative study of aberrant biliary vesicles in man and the higher mammals. Anat. Rec..

[11] Harlaftis N., Gray S.W., Skandalakis J.E. (1977). Multiple gallbladders. Surg. Gynecol. Obstet..

[12] Bayerdörffer E., Ritter M., Hatz R., Brooks W., Ruckdeschel G., Stolte M. (1994). Healing of protein losing hypertrophic gastropathy by eradication of Helicobacter pylori—Is Helicobacter pylori a pathogenic factor in Ménétrier’s disease?. Gut.

[13] Alcántara-Figueroa C.E., Calderón-Cabrera D.C., Pariona-Martínez Y.K., de la Cruz-Rojas R., Alcántara-Ascón R.A. (2023). Ménétrier disease: A rare cause of hypertrophic gastropathy. Rev. Gastroenterol. Mex..

[14] Shi W., Jiao Y. (2023). Ménétrier’s disease. QJM.

[15] Zhang T., Chen T., Tang X. (2026). Ménétrier’s disease: A narrative review of molecular pathogenesis, clinical spectrum and evolving therapeutic strategies. QJM.

[16] Xiao S.Y., Hart J. (2001). Marked gastric foveolar hyperplasia associated with active cytomegalovirus infection. Am. J. Gastroenterol..

[17] Huh W.J., Coffey R.J., Washington M.K. (2016). Ménétrier’s Disease: Its Mimickers and Pathogenesis. J. Pathol. Transl. Med..

[18] Nguyen V.X., Nguyen C.C., Leighton J.A., Pasha S.F., Silva A.C., Heppell J.P., De Petris G. (2010). The Association of Ménétrier Disease with Ulcerative Colitis: A Case Report with Implications on the Pathogenesis of Ménétrier Disease. Case Rep. Gastroenterol..

[19] Wu J., Chen Y., Yin J., Li P., Hu H. (2025). Ménétrier Disease: Rare Manifestations and Mechanistic Insights From 2 Adult Cases. Am. J. Case Rep..

[20] Aamar A., Madhani K., Virk H., Butt Z. (2016). Zollinger-Ellison syndrome: A rare case of chronic diarrhea. Gastroenterol. Res..

[21] Yip R.H.L., Lee L.H., Lee L.H., Schaeffer D.F., Horst B.A., Yang H.-M. (2020). Topography, morphology, and etiology of lymphocytic gastritis: A single institution experience. Virchows Arch..

[22] Ahn J.Y. (2026). Clinical management of gastric mucosa-associated lymphoid tissue lymphoma. Korean J. Intern. Med..

[23] Leonard N.B., Bronner M.P. (2021). Giant Gastric Folds in Juvenile Polyposis. Case Rep. Gastroenterol..

[24] Bernshteyn M., Bhutta A.Q., Bordas J., Mehta R., Arif M.O. (2022). A Rare Case of Juvenile Polyposis Syndrome Mimicking Ménétrier’s Disease. Cureus.

[25] Keener M., Waack A., Ranabothu M., Ranabothu A., Vattipally V.R. (2023). Clinical and Radiological Features of Menetrier’s Disease: A Case Report and Review of the Literature. Cureus.

[26] Pepa P., Uehara T., Wonaga A., Redondo A., Avagnina A., Mazzocchi O., Antelo P., Waldbaum C., Sorda J. (2021). Menetrier’s disease. A diagnostic and therapeutic challenge. Medicina.

[27] Snoubar I., Alhendi T., Batanji D., Nabresi N., Sultan M., Bannoura S., Einar K. (2025). Pediatric Ménétrier’s Disease Triggered by *Cytomegalovirus* Infection: A Rare Case of Severe Hypoalbuminemia and Edema. Case Rep. Pediatr..

[28] Serena P., Mare R., Miutescu B., Bende R., Popa A., Aragona G., Seclăman E., Serena L., Barbulescu A., Sirli R. (2025). Resistance to Clarithromycin and Fluoroquinolones in *Helicobacter pylori* Isolates: A Prospective Molecular Analysis in Western Romania. Antibiotics.

[29] Chey W.D., Howden C.W., Moss S.F., Morgan D.R., Greer K.B., Grover S., Shah S.C. (2024). ACG Clinical Guideline: Treatment of Helicobacter pylori Infection. Am. J. Gastroenterol..

[30] Almazar A.E., Penfield J.D., Saito Y.A., Talley N.J. (2021). Survival Times of Patients with Menetrier’s Disease and Risk of Gastric Cancer. Clin. Gastroenterol. Hepatol..

[31] Khan A., Chhaparia A., Hammami M.B., Hachem C. (2020). Role of Octreotide in Menetrier’s Disease: Case Report and Review of Literature. Cureus.

[32] Fiske W.H., Tanksley J., Nam K.T., Goldenring J.R., Slebos R.J., Liebler D.C., Abtahi A.M., La Fleur B., Ayers G.D., Lind C.D. (2009). Efficacy of cetuximab in the treatment of Menetrier’s disease. Sci. Transl. Med..

[33] Ramos-Morales P., Villegas C., Verdines-Perez A.M., Alvarez-Lozada L.A., Valdivia-Balderas J.M., Elizondo-Omaña R.E., Quiroga-Garza A. (2026). Gallbladder Duplication Diagnostic and Surgical Implications: Case Report and Classification Synthesis. Surg. Case Rep..

[34] Ferreira C., Noel C.B. (2021). Biliary tract anatomical variance—The value of MRCP. S. Afr. J. Surg..

[35] Liu X., Mu J., Pang M., Fan X., Zhou Z., Guo F., Yu K., Yu H., Ming D. (2024). A Male Patient with Hydrocephalus via Multimodality Diagnostic Approaches: A Case Report. Cyborg Bionic Syst..

